# Glutamine Synthetase as an Astrocytic Marker: Its Cell Type and Vesicle Localization

**DOI:** 10.3389/fendo.2013.00144

**Published:** 2013-10-16

**Authors:** Enrico Anlauf, Amin Derouiche

**Affiliations:** ^1^Institute of Anatomy II, University of Frankfurt, Frankfurt am Main, Germany; ^2^Dr. Senckenbergisches Chronomedizinisches Institut, University of Frankfurt, Frankfurt am Main, Germany

**Keywords:** astrocyte, oligodendrocyte, glutamate metabolism, immunocytochemistry, deconvolution

The overall staining by GS clearly reveals astrocytes, including all cells of the astroglial family (1), i.e., Bergmann glia, Müller cells (2), tanycytes (3), and ependymal cells. The star shaped morphology from classical silver impregnations relates to cortical and hippocampal astrocytes, which display a comparable pattern in material stained for GFAP. However, the dense population of GS stained astrocytes found in all diencephalic and mesencephalic regions, known to display faint GFAP-labeling (unpublished observations) indicated that while apparently all astrocytes contain GS they have GFAP-ir filaments only in a region-dependent pattern. This is complicated by the emerging view that “astrocytes” constitute a heterogeneous population even within a given region. In the rat hippocampus, combined immunostainings have revealed that the “classical” GFAP-ir astrocyte constitutes a subpopulation of GS-ir astrocytes, which can also lack GFAP staining [direct double staining (4)]. In view of several astroglial subtypes and/or glial precursors present in the adult rodent brain, anti-GS appears to be the most general astrocyte marker, covering all subtypes. In addition, GS has been found early on to label exclusively astrocytic cells and no other glial or neuronal cell types *in situ* or in culture [reviewed by (5)]. GS has, thus, been applied as a reliable astrocyte marker in very many studies since.

“Complex cells” in rat hippocampus, initially assumed to be an astrocyte subtype (6) but now understood to belong to NG2 cells, a fourth glial type in the CNS (7), may display faint GS-ir in the soma but not its fine processes. Oligodendrocyte precursor cells, possibly also related to NG2 cells, were found to be devoid of GS-ir (8). The immunocytochemical profile and possible heterogeneity of NG2 cells is still under debate to date. Disputing the exclusion of non-astrocytic cells in GS staining, some authors have later reported GS^+^ oligodendrocytes, although this has not been investigated systematically. Reports on non-astrocytic GS will be discussed in detail here.

## Oligodendrocytes

The authors observing oligodendroglial GS localization rely mostly on the non-convincing morphology of “ovoid cells” in the gray matter, and only sometimes on the unambiguous alignment of interfascicular oligodendrocytes. Only one study is based on GS mRNA *in situ* hybridization (GS exclusively in astrocytes), and three studies on colocalization of GS-ir with oligodendroglial markers (see below). The reports on GS-ir in oligodendrocytes by three groups (9–11) can, however, not be reconciled, and might result from the use of different antisera and/or divergent interpretations of morphology. Thus, Cammer (9), applying a proprietary anti-sheep brain GS, observed clearly intrafascicular oligodendrocytes, but only faint white matter astrocytes in rat spinal chord. A similar pattern was evident in rat forebrain white matter (proprietary GS antiserum; specimens prepared by Dr. M. Lavialle). Anti rat liver GS (9) produced the most convincing intrafascicular oligodendrocytes displaying also immunoreactivity for CNPase, an established oligodendrocyte marker. However, in gray matter, oligodendrocytes but hardly astrocytes were detectable by anti-GS. Based on a different rabbit anti-sheep brain GS antiserum in cat brain, GS localization was found in an inverse relation, i.e., only in gray matter oligodendrocytes but not interfascicular oligodendrocytes (10). These cells were identified by light microscopic morphological criteria, most of them in perineuronal position. A localization of GS in gray (but not white) matter oligodendrocytes, mostly perineuronal and perivascular was confirmed by plausible ultrastructural criteria, using another rabbit anti-sheep brain GS in the mouse brain (11). In this context, the absence of a typical light microscopical pattern distinguishing astrocytes from oligodendrocytes in perineuronal position in cortex or hippocampus, and generally in non-telencephalic regions (where astrocytes are generally non-stellate) might be relevant. In these regions, the GS-ir gray matter oligodendrocytes observed by Miyake and Kitamura (11) were particularly abundant but “astrocytes” were hardly observed. GS-ir perineuronal oligodendrocytes were present in addition to astrocytes also in the cortex but not in the hippocampus, which would imply subclasses of perineuronal oligodendrocytes. Similarly, the figures provided by (10), of GS-ir gray matter oligodendrocytes in the cortex (perineuronal) and cerebellum (around Purkinje cells,) do not allow for clear differentiation from astrocytes or Bergmann glia. This applies particularly to the cerebellum where even an “oligodendrocyte-like astrocyte” has been described (12). In contrast, the GS-ir “ovoid cells” in the lizard mesencephalon were interpreted as astrocytic, since they were in alignment with radial glial fibers, and forming perivascular end feet (13). Non-astrocytic labeling by anti-GS might also be associated with technical difficulties. Thus, Werner et al. (14), although colocalizing GS with CNPase in oligodendrocytes, depict GS-ir in the nucleus or putative perinuclear cytoplasm, but not in processes. They also find positive microglia, which has never been reported before, and would normally represent a negative control. Similarly, in the report on GS staining in over 50% of CNPase positive perineuronal oligodendrocytes (15), the GS-staining was not seen within the typical, ring-like CNPase^+^ cell rim (as clearly shown for several other markers in the same publication). Although applying confocal microscopy, the images show occasional 3D overlay of incongruent shapes in the two channels, which might lead to misinterpretation particularly since the authors did not consider neighboring, perineuronal astrocytes. In cell culture, where glial cell type purity and technical preparation may lead to diverging results, GS can be induced in cells that are normally not GS^+^, such as fibroblasts (16) or even chick brain neurons, which are GS^−^ negative *in vivo* (17). GS induction in cultured oligodendrocytes, by corticoids or thyroid hormones was observed by some (18) but not others (17).

As evidence in favor of an astrocyte-restricted GS localization [reviewed by (5)], absence of oligodendrocyte labeling in white and gray matter has been reported by the group of Norenberg, in particular at the ultrastructural level (19, 20), and the group of Derouiche, who investigated vibratome sections from human (21), and rat (3), using a previously characterized anti-GS antiserum (16) or commercial GS antibodies (Chemicon-Millipore, Billerica, MA, USA: clone GS-6, MAB 302; Santa Cruz Biotechnology, CA, USA, Ab: sc-6640; unpublished observations). In particular, GS-ir in the conspicuous interfascicular oligodendrocytes would not have been overlooked in the studies mentioned, and very many others. Based on distribution and morphology, localization of GS mRNA in rat brain, although without cell identification, was in line with exclusive astrocytic labeling (22).

The clear distinction by GS-ir between astroglial cells and oligodendrocytes would be maintained in the tumors derived from these cells, since all astrocytomas and ependymomas but none of the oligodendrogliomas were GS^+^ (23). Another astrocytic antigen, ezrin, which labels predominantly the fine, peripheral astrocyte processes of all astrocytic cells but not oligodendrocytes (24), has a corresponding, clear-cut specificity within the range of human glial tumors (25).

## Neurons

Neuronal localization of GS has been undisputedly excluded by all studies, apart from two reports on human autoptic material from normal subjects, and individuals suffering from Alzheimer’s disease (26, 27). In addition to astrocytes, the perikarya particularly of pyramidal cells were intensely labeled. Labeled neurons were observed in 2 (of 7) normal brains, and were highly variable in localization and quantity over cortical fields and layers also in all 10 cases from Alzheimer demented subjects (27). However, previous evidence and technical considerations suggest that these observations should be taken *cum grano salis*. Although there is no animal model for Alzheimer’s disease, neuronal localization has not been reported in any of the many experimental neuropathology studies employing GS-ir. The finding also contrasts with previous human data (21) (4 cases), (23) (15), (28) (17). The only neuronal localization of GS *in situ* has been reported in a proteomic analysis of squid optic lobe synaptosomes, a definitely glia-free preparation (29). However, neuronal labeling similar to that reported (26, 27) has been observed in sections from rat brain (perfusion or immersion-fixed, vibratome) or human hippocampus (vibratome, paraffin), applying various anti-GS antisera in two laboratories (author’s unpublished observations; Dr. M. Lavialle, personal communication). This neuronal labeling was regarded as spurious, since it occurred inconsistently after storage (exceeding 1 week), often without the expected glial staining, even in vibratome sections from the same block that has yielded the exclusive astroglial pattern in staining runs before. This might indicate the recognition of distinct epitopes displaying independent physicochemical properties. Interestingly, the anti-GS mAb (Chemicon) also used by (27, 28) has been found to cross reacts with a “GS-like protein” different from GS (30), however, its cellular localization in the brain has not been established.

## Subcellular GS Localization

**Figure 1:**
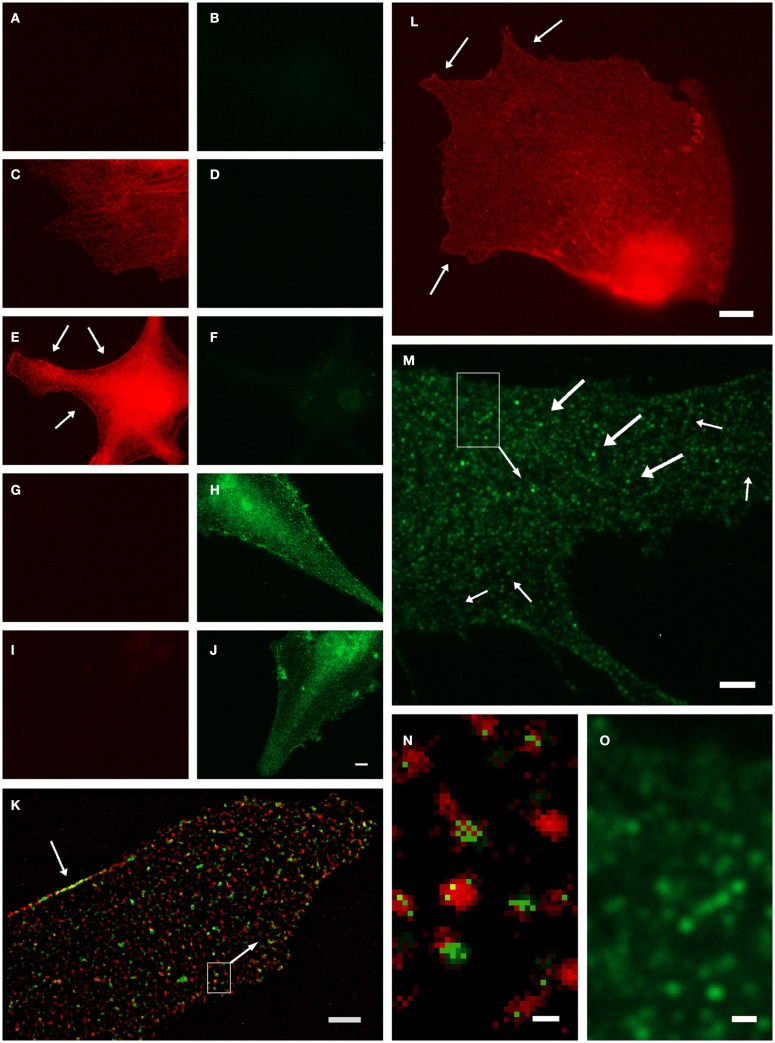
**Organelle-bound localization of GS-immunoreactivity, in primary culture of rat cortical astrocytes using the following anti-GS antibodies: (1) polyclonal made in rabbit (15), or (2) goat (Santa Cruz sc-6640), or (3) mouse monoclonal (Chemicon-Millipore, Billerica, MA, USA; clone GS-6, MAB 302)**. **(A–J)** Controls for double-labeling (red and green) with two anti-GS antibodies, red channel always in left, green in right column. **(A,B)** Control for autofluorescence, no immunoreagents. **(C,D)** Control for fluorescence red-to-green bleed through: Single staining anti-GS (1) with secondary anti-rabbit antibody (red). **(E,F)** Control for detection system of green channel: Same as in **(C,D)**, in addition secondary anti-mouse antibody (green). **(G,H)** Control for fluorescence green-to-red bleed through: Single staining anti-GS (3) with secondary anti-mouse antibody (green). **(I,J)** control for detection system of red channel: Same as in **(G,H)**, in addition anti-rabbit antibody (red). **(K)** Double-labeling by antibodies (1) and (2) coincides on the same organelles, even at high magnification [**(N)**, from inset in **(K)**]. Antibody (1), red channel, labels the complete outline of organelles, whereas antibody (2), green channel only yields pixels within the extent of individual red labeled structures. Optical section 100 μm thick, after deconvolution. Single labeling by antibodies (3) **(L)** or (2) [**(M,O)**, from inset] yields comparable organelles. Scale 5 μm [in **(J)**, for **(A–J)**], 3 μm **(K,M)**, 4 μm **(L)**, 0.25 μm **(N)**, 0.5 μm **(O)**.

Anti-GS has been found to represent an ultrastructural marker completely “filling” astrocytic cytoplasm *in situ*, well suited to mark also the extremely fine glial processes (20, 31). However, it was noted that labeling might also be associated with vesicles (5), which could not be verified in the stainings based on the diffusible chromogen DAB (5). We further investigated this applying high-resolution fluorescence microscopy and deconvolution in primary astrocyte culture (32). Based on negative controls (Figures 1A–J), GS-ir using a polyclonal antibody made in rabbit (16) was concentrated in discrete structures resembling vesicles (Figures 1K–O), which were also double-labeled applying different GS antibodies simultaneously (Figures 1K,N). Note that there is no full “colocalization,” i.e., full pixel superimposition of green and red channels. With the individual organelle as the unit of observation, however, the sparse, mostly incoherent pixels of the green channel [goat anti-GS (Santa Cruz sc-6640)] are mostly associated with the discrete, vesicle-like structures of the red channel (Figures 1K,N). This finding, possibly resulting from differential labeling efficiencies of the antibodies, can be referred to as vesicular colocalization. These double-labeled vesicular structures were present throughout the cell but concentrated at limited stretches of the cell boundaries (arrows in Figures 1K,L, also in Figures 1E,H,J), they were frequently arranged in rows (arrows in Figures 1M,O), and their structure as far as could be resolved was non-uniform, pleomorph (Figures 1M,O).

While these observations do not necessarily exclude the commonly assumed cytosolic presence of GS, they clearly suggest a vesicle-bound form. GS might be indirectly linked to vesicular membranes, as is known, e.g., from the glutamic acid decarboxylase isoform GAD65, which is attached to transmitter vesicles via the vesicular GABA transporter, to support neurotransmission (33). It will be interesting to further investigate whether these findings are important in the context of metabolic compartmentation, in particular in relation to glial glutamate uptake, metabolism, and release (34). Similarly, how would the GS^+^ vesicles relate to those involved in vesicular exocytosis of glutamate from astrocytes?
